# In Situ Cytokine Expression, Nitric Oxide and Reactive Oxygen Species Production During Primary Amoebic Meningoencephalitis Development

**DOI:** 10.3390/ijms27198693

**Published:** 2026-09-29

**Authors:** Moisés Martínez-Castillo, Verónica Ivonne Hernández-Ramírez, Patricia Talamás-Rohana, Itzel Citlalli Rubio-Gutiérrez, Mineko Shibayama, Jesús Serrano-Luna

**Affiliations:** 1 Departamento de Hígado Páncreas y Motilidad (HIPAM), Unidad de Medicina Experimental, Escuela de Medicina de la Universidad Nacional Autónoma de México, Hospital General de México, Dr. Balmis 148 Colonia Doctores, Mexico City 06726, Mexico; 2 Departamento de Infectómica y Patogénesis Molecular, Centro de Investigación y de Estudios Avanzados del Instituto Politécnico Nacional, Av. IPN 2508, Mexico City 07360, Mexico; 3 Departamento de Biología Celular, Centro de Investigación y de Estudios Avanzados del Instituto Politécnico Nacional, Av. IPN 2508, Mexico City 07360, Mexico

**Keywords:** *Naegleria fowleri*, primary amoebic meningoencephalitis, inflammatory cytokines, nitric oxide, reactive oxygen species

## Abstract

Primary amoebic meningoencephalitis (PAM), caused by *Naegleria fowleri*, is a rapidly progressive and fatal infection, and inflammation of the olfactory bulb plays a key role in disease pathogenesis. Although inflammatory and oxidative mediators have been studied in vitro, their production during the progression of PAM remains poorly understood. This study evaluated the temporal production of TNF-α, IL-1β, IL-6, nitric oxide (NO), and reactive oxygen species (ROS) in the olfactory bulbs of BALB/c mice from 1 to 7 days after intranasal infection with *N. fowleri* trophozoites. RT-PCR detected increased Tnf, Il1b, and Il6 transcript levels from day 3 post-infection. Western blot analysis complemented these findings by detecting the corresponding cytokine proteins from day 2, although their temporal patterns differed among the cytokines examined. Immunohistochemical analysis demonstrated that cytokine production was associated with the presence and accumulation of inflammatory cells in the olfactory bulb. In addition, ROS and NO production increased throughout disease progression, indicating the establishment of a pronounced oxidative microenvironment. These findings demonstrate that *N. fowleri* infection induces a sustained local inflammatory response characterized by cytokine overproduction and oxidative stress. Together with inflammatory cell infiltration, these mediators may contribute to extensive olfactory bulb damage and disease severity, ultimately leading to host death.

## 1. Introduction

*Naegleria fowleri* is a free-living ameboflagellate that has been isolated from soil and water worldwide. This microorganism is the etiologic agent of primary amoebic meningoencephalitis (PAM), an acute and fatal disease that affects the central nervous system (CNS) [1]. This infection has been reported mainly in children and young adults; their aquatic activities in contaminated freshwater bodies were recently identified as a vector of transmission [2]. Unfortunately, patient death generally occurs approximately one week after symptoms appear (95% of cases). Cases of recovery have occurred, associated with prompt diagnosis and the use of amphotericin B; however, to date, there is no specific drug available against PAM [3].

Brain histopathology from human biopsies usually reveals the presence of amoebae, necrosis, and intense inflammatory reactions, mainly in the olfactory bulb (OB) [2,4]. Moreover, the OB presents disarrangement, with hemorrhage and neutrophils scattered in the necrotic areas, accompanied by meningeal exudates showing equal proportions of neutrophils and chronic inflammatory cells [4,5]. Mice, and more recently, rats, have been excellent models for studying the different stages of PAM because they experience histopathological events that resemble those in human cases [6,7,8,9]. In the late stages of PAM, the brains of these infected animals display an intense inflammatory response characterized by the presence of abundant neutrophils and macrophages, which are associated with lytic brain necrosis and hemorrhage [6,9,10,11]. Moreover, CD38−/− knockout mice (deficient in neutrophil chemotaxis) infected with *N. fowleri* developed PAM but were more likely to survive than the wild-type mouse strain (C57BL/6J). Therefore, the authors conclude that the exacerbated inflammatory reaction plays an important role in PAM progression [9].

Interestingly, in vitro studies have reported that *N. fowleri* trophozoites can damage neuroblastoma cells [12,13]. Moreover, live trophozoites and their excretory and secretory proteins can induce the overexpression of proinflammatory cytokines (TNF-α, IL-1α, IL-1β, and IL-6) in astrocytes and microglia through the AP-1 and NF-kB signaling pathways [14]. Recently, it was reported that *N. fowleri* could induce the expression of VCAM-1 and ICAM-1 and the production of IL-8, IL-1β, TNF-α, and IL-6, as well as nitric oxide, in a primary culture of endothelial cells [6]. Therefore, the authors suggest that the production of cytokines by microglia and endothelial cells could have contributed to the intense inflammatory response and migration of inflammatory cells to the OB during PAM infection [6,14].

In the brain, both microglia and astrocytes can produce important concentrations of nitric oxide (NO) and reactive oxygen species (ROS) [15], and these radicals are essential for the control and resolution of the inflammatory process; however, excessive production leads to oxidative stress, which is instrumental in the progression of microbial infections and neurodegenerative diseases [16,17,18]. In vitro studies reveal that NO production in murine macrophages is essential for killing *N. fowleri* trophozoites through the L-arginine-dependent pathway [19]. In addition, Rojas-Hernández et al. suggested that *N. fowleri* contains specific nitric oxide synthase (NOS) isoforms that may offer it biochemical mechanisms to proliferate and survive under oxidative conditions [20]. It was also reported that human neutrophils could kill *N. fowleri* trophozoites through the release of neutrophil extracellular traps (NETs) in in vitro systems. The authors reported that myeloperoxidase and neutrophil elastase present in NETs participated in the successful elimination of *N. fowleri* trophozoites [21]. Furthermore, the efficient activation of neutrophils is reported to occur via the hydrogen/myeloperoxidase/halide system, which is a key regulator of ROS production [22]. Nevertheless, until now, a detailed experimental study of the role of NO and ROS in olfactory bulb damage during PAM has not been performed.

In the present study, we evaluated the role of inflammation in the OB in situ by analyzing the expression and production of inflammatory cytokines, as well as ROS and NO levels, during the evolution of PAM. We observed that *N. fowleri* trophozoites induce the expression and production of TNF-α, IL-1β, and IL-6 in a time-dependent manner during PAM development, which is directly correlated with the arrival of inflammatory cells. Moreover, we found that the presence of trophozoites causes increases in NO and ROS concentrations in the OBs of infected animals. Therefore, our results strongly suggest that the inflammatory process and oxidative stress microenvironment begin in the OBs and participate in tissue damage during the evolution of PAM, ultimately leading to the death of the host.

## 2. Results

### 2.1. N. fowleri Induces Proinflammatory Cytokine Expression During the Evolution of PAM

To examine the local immune response induced by *N. fowleri* in the OB, we evaluated whether transcripts of different inflammatory cytokines were present from one to seven days post-infection. The results revealed that the expression of TNF, IL-1β, and IL-6 in infected mice increased in a time-dependent manner beginning on the third day post-infection, with RNA expression peaking on the seventh day post-infection. All the proinflammatory cytokines evaluated exhibited the same behavior (Figure 1A). In contrast, we did not find significant differences in the cytokine levels of healthy animals at seven days. Densitometric analysis of the mRNA expression of each cytokine product was normalized to that of the housekeeping gene *Gapdh*, and the OD values were plotted vs. infection days (* *p* < 0.05; ** *p* < 0.01) (Figure 1B).

### 2.2. In Situ TNF-α, IL-1β, and IL-6 Production in the Mouse Brain During Progression of PAM

The results revealed that cytokine-associated signals were detectable from day 2 post-infection and exhibited distinct temporal patterns. Changes were mainly observed for IL-1β from days 2 to 6 post-infection (Figure 2A). Cytokine levels were assessed up to day 6 post-infection, when the animals showed signs of severe disease and the tissue became highly friable. TNF-α was detectable from days 2 to 6, with a more pronounced signal on days 5 and 6. IL-6 levels were lower than those of the other cytokines, although its signal was also detectable from days 2 to 6 and varied among the evaluated time points. Densitometric analysis of TNF-α, IL-1β, and IL-6 signals on days 3, 4, 5, and 6, normalized to the corresponding β-actin signal, was broadly consistent with the mRNA expression results obtained from day 3 post-infection. Noninfected mice showed no detectable signals for the cytokines examined. Three independent assays yielded similar patterns (Figure 2B; *p* < 0.05).

The results of the immunohistochemistry assays revealed a slight inflammatory reaction on the fourth day post-infection; neutrophils and macrophage infiltration were positive for TNF-α, IL-1β, and IL-6 (Figure 3). On the fifth day post-infection, neutrophils and macrophages in proximity to trophozoites were positive for TNF-α. Some areas with necrotic tissue were observed at this time (Figure 3). A similar pattern of labeling was observed for IL-1β and IL-6. After six days of infection with *N. fowleri*, the inflammatory infiltrates were positive for all the cytokines analyzed. In addition, OB, necrosis, and a large number of amoebae mixed with inflammatory cells were observed (Figure 3).

### 2.3. Nitric Oxide (NO) Levels Increase During MAP Evolution

Nitric oxide is a signaling molecule that plays a key role during inflammation. For this reason, we decided to evaluate the NO levels produced in the OB during PAM evolution by determining the total product nitrite/nitrate (NO_2_ + NO_3_). The results revealed a significant increase in the concentration of NO products after the third day post-infection (*p* < 0.05); however, higher levels were reached at six and seven days, with a peak at the seventh day post-infection that was 26 times greater than that in the control group (*** *p* < 0.001). These significant increases in NO levels could be a consequence of neutrophil and macrophage infiltration in the infected OBs (Figure 4).

### 2.4. Reactive Oxygen Species Were Produced Locally During N. fowleri Infection

ROS-induced oxidative stress significantly influences tissue damage during microbial infection. To determine ROS production during experimental PAM, the brains of infected animals from each infection day were processed to measure oxidative stress and compare them to those of control animals (healthy animals). Compared with those in the control group, the ROS levels in the infection group were significantly greater at 5, 6, and 7 days after infection (* *p* < 0.05, ** *p* < 0.01) (Figure 5). These results correlated with the excessive inflammatory response induced by *N. fowleri* infection. The tissues treated with H_2_O_2_ (positive control) exhibited maximal ROS production (*** *p* < 0.001).

## 3. Discussion

Pathogenic *N. fowleri* produces PAM, and this acute and fatal illness usually causes the death of the human host from the third to the tenth day post-infection [23]. Although the CNS is considered an immune-privileged organ, some pathological infections or traumatic events promote the production of cytokines, complement, NO, and ROS in the brain [24]. Furthermore, the characteristic exacerbated immune response and its correlation with tissue injury have been explored in both the presence and absence of pathogens [25,26,27]; experimental PAM has been used in mice to analyze immunopathological events during disease progression [9]. The initial stages of infection in the nasal cavity at the neuroepithelium region (6–12 h) are characterized mainly by the presence of neutrophils surrounding *N. fowleri* trophozoites [10]. However, trophozoites can evade this innate immune response in the olfactory mucosa and migrate until they reach the OBs three days post-infection [9]. The number of trophozoites increases with the concomitant presence of eosinophils and neutrophils in the OBs. In addition, after four to seven days of infection, areas of lytic necrosis and hemorrhage are evident [9]. The role of the inflammatory process in N. fowleri-induced death has been evaluated in CD38−/− knockout mice (animals deficient in neutrophil recruitment); interestingly, the log-rank test and survival curves revealed that compared with the wild-type strain, the infected knockout animals survived longer. Although an inflammatory reaction is usually a protective mechanism to eliminate pathogens from host tissues [28], the authors demonstrated that the host inflammatory response against N. fowleri, mainly the activation of polymorphonuclear cells with subsequent lysis, contributes to CNS tissue damage [9]. The regulation of this response is mediated by soluble factors, including cytokines, interferons, tumor necrosis factor (TNF), chemokines, and growth factors [29]. Some in vitro studies have been carried out to analyze the production of inflammatory cytokines stimulated by *N. fowleri* [6,30,31,32,33], but their production in the brain has not been described until now. Here, we observed a significant increase in the expression of cytokines and the production of TNF-α, IL-6, and IL-1β in OBs at three days post-infection, while immunohistochemical analysis revealed clear labeling of the inflammatory cells for TNF-α, IL-6, and IL-1β at four days post-infection and a clear increase in the production of these cytokines at five to six days. Western blot analysis further demonstrated that TNF-α, IL-1β, and IL-6 proteins were detectable as early as day 2 post-infection, preceding the increase in their transcript levels detected from day 3. These findings indicate that the local inflammatory response is initiated early during infection and progressively increases as PAM develops, although the temporal pattern of cytokine production differs among the cytokines examined. It seems that neurons, glia, and endothelial cells can express TNF-α, IL-6, and IL-1β in the CNS [34]. In fact, in the brain, microglia are the first cells to recognize foreign molecules and pathogens and consequently produce IL-8, IL-1, IL-3, IL-6, IL-10, and TNF-α, among other immune mediators [35,36,37]. Moreover, in an in vitro system, *N. fowleri* co-incubated with microglia and astrocytes expresses TNF-α, IL-1β, and IL-6 [30,32]. In a more recent study, it was shown that the cathepsin Bs of *N. fowleri* (Nf-CBs) can initiate proinflammatory responses in BV-2 microglia via the MyD88-dependent TLR-2/TLR-4 pathway and that the increased expression of proinflammatory cytokines such as TNF-α, IL-α, IL-1β, and IL-6 is mediated by NF-κB and the AP-1-dependent MAPK signaling pathway. Therefore, the authors concluded that Nf-CB might contribute to the pathogenesis of PAM by inducing a proinflammatory immune response in microglia via a contact-independent pathway, promoting a subsequent inflammatory cascade in the brain [33]. It is possible that in vivo, glial cells (microglia and astrocytes) recognize soluble factors released by *N. fowleri* and contribute to the production of cytokines. At present, microglial cell culture can be activated by molecules released from *N. fowleri* in a contact-independent manner [38]. Our results revealed that inflammatory cells (neutrophils and some macrophages) were the principal positive lineages for cytokine production. Future studies using specific markers are necessary to differentiate other types of cells involved in the production of TNF-α, IL-6, and IL-1β.

We observed that inflammation increased over time, which was associated with damage to and necrosis of the olfactory bulbs. We also detected increases in NO and ROS production. It is known that inducible NO has antipathogenic and antitumoral properties [39]; however, overproduction can induce toxic effects in brain cells [15,16,40]. In vitro, studies have shown that NO production is involved in the destruction of *N. fowleri* trophozoites by activated macrophages [19]. Our results revealed tissue damage and necrosis of the OBs, which correlated with the increase in NO levels. Interestingly, Rojas-Hernández et al. (2007) reported an ortholog of nitric oxide synthase in *N. fowleri* trophozoites, suggesting the production of NO by the amoeba [20]; thus, these cellular mechanisms may allow the survival of *N. fowleri* trophozoites under high-NO conditions. In addition, the interaction of amoebae with rat brain microvascular endothelial cells (RBMECs) induces the production of NO, promoting dysfunction of the blood–brain barrier and allowing the arrival of inflammatory cells to OBs [6]. In this inflammatory microenvironment, the recruitment of neutrophils to the brain may be due to the systemic and local production of proinflammatory cytokines, and NO is a consequence of trying to eliminate amoebae. Neutrophils present a wide battery of mechanisms against microorganisms, including degranulation and the production of proteolytic enzymes, antimicrobial peptides, and reactive oxygen species (ROS), among others [41,42]. Neutrophils can increase oxygen uptake to generate ROS to kill invading pathogens. It has been reported in vitro that activated neutrophils are able to kill *N. fowleri* trophozoites through neutrophil extracellular traps (NETs) and reactive oxygen species such as myeloperoxidase [21,22]. However, in advanced stages of experimental PAM, *N. fowleri* is capable of dividing significantly; thus, this suggests that the amoeba resists the negative effects of diverse ROS, and *N. fowleri* can also induce ROS-dependent programmed cell death, necroptosis, in Jurkat T cells [43]. In *Naegleria gruberi*, a nonpathogenic species, some predicted proteins and enzymes that allow the survival of trophozoites under both aerobic and anaerobic conditions have been reported through genomic analysis [44].

Among the predicted molecules that can participate in resistance against the ROS microenvironment in *N. gruberi*, the peroxisomal targeting signal at the C-terminus (PTS1) and peroxins (PEX) are proteins involved in the biogenesis of peroxisomes. Authors have also suggested that alternative oxidase (AOX), which is an enzyme that can control the levels of superoxide, nitric oxide, and other important redox couples [45], can be essential during the proliferation of *N. fowleri* in the brain [44]. Recently, the antirheumatic drug Aurofin was shown to have good amoebicidal effects in vitro assays. This drug can bind to and inhibit thioredoxin–glutathione reductase (TrxR), causing the dysregulation of cellular redox control in Schistosoma mansoni; moreover, this drug has antimicrobial effects on *Giardia lamblia* and *Entamoeba histolytica* [46]. Some of the specific roles of Prx include the regulation of reactive sulfur species (such as RS, RSSR→, or RSOOH), as well as the control of ROS such as O_2_→, H_2_O_2_, or ROOH [47]. Taken together, these mechanisms may allow *N. fowleri* to survive despite oxidative stress conditions during the evolution of PAM. Unfortunately, the extensive inflammatory response during microbial infections induces concomitant tissue damage [16,17,18,48]. Neutrophil-induced damage in inflamed organs is widely reported; this effect is related to the overproduction of NO, ROS, proteolytic enzymes, and cytokines [41,42,49,50]. For example, exogenous TNF-α administration results in dose-dependent cytotoxicity in primary septo-hippocampal cultures [51]. In addition, the intracerebral injection of recombinant TNF-α or IL-1β markedly exacerbates ischemic and cytotoxic injury in vivo [34]. Moreover, it has been reported in mice that the overexpression of IL-6 results in marked neurodegeneration through direct neurotoxic effects [52]. As mentioned above, neutrophils play important roles in the deaths of infected animals [9]; however, the presence of these cells in addition to NO and ROS in the OBs of infected mice and their direct role in animal death have not been demonstrated until now. Overall, our study provides in vivo evidence that inflammation and the oxidative environment are important factors in PAM evolution due to imbalanced cytokine levels and NO and ROS overproduction in the brain.

## 4. Materials and Methods

### 4.1. Amoebic Cultures

*Naegleria fowleri* trophozoites (ATCC 30808) were maintained axenically in 2% Bactocasitone medium (Thermo Fisher Scientific, Waltham, MA, USA) supplemented with 10% fetal bovine serum (FBS) (Equitech-Bio, Inc., Kerrville, TX, USA) at 37 °C. For mouse instillation, the trophozoites were harvested during the logarithmic phase of growth (48 h).

### 4.2. Experimental Animals

Four-week-old male BALB/c mice were anesthetized with an anesthetic mixture composed of tiletamine/zolazepam (Zoletil^®^ Virbac S.A. Carros Cedex, France) at a dosage of 20 mg/kg and xylazine (Virbac, Zapopan Jalisco, Mexico) at 15 mg/kg via intraperitoneal injection. Afterward, the animals were inoculated nasally with 20 μL of fresh amoebic culture medium without FBS containing 2.5 × 10^4^ trophozoites. After different post-inoculation (PI) periods (1 to 7 days), the animals’ brains, containing the olfactory bulb (OB), were dissected and processed for RT–PCR, Western blotting (WB), and immunohistochemistry to determine the expression and production of proinflammatory cytokines. Furthermore, the OBs were used to determine NO and ROS production during the evolution of PAM. As an experimental control, non-infected animals were maintained under the same conditions until the maximum time of study. All the animals were handled in accordance with Mexican federal regulations for animal experimentation and care (NOM-062-ZOO-1999), and the study was approved by the Institutional Animal Care and Use Committee (IACUC-CINVESTAV). The mice were humanely euthanized by an overdose of an anesthetic mixture composed of tiletamine/zolazepam (Zoletil^®^), and the brains were collected after death was confirmed for further analyses.

### 4.3. Analysis of Cytokine Expression by RT–PCR

To determine the expression levels of cytokines in situ, total RNA was extracted from the affected parts of the mouse brains (olfactory bulbs) at different times during PAM progression. The expression of each cytokine was compared with that in samples from healthy mouse brains. Briefly, approximately 100 mg of brain tissue containing OB was homogenized with 1 mL of TRIzol reagent (Life Technologies, Carlsbad, CA, USA). After overnight precipitation at −20 °C, the mixture was centrifuged at 12,000× *g* for 15 min; the aqueous phase was recovered, and an equal volume of isopropanol (0.5 mL) (Sigma-Aldrich, St. Louis, MO, USA) was added to obtain the mRNA. The mRNA was amplified using a OneStep RT–PCR kit according to the manufacturer’s instructions (Qiagen, Hilden, NRW, Germany).

The primers used to amplify proinflammatory cytokines were as follows: *TNF*- was amplified with the sense primer 5′-TTGACCTCAGCGCTGAGTTG-3′ and antisense primer 5′-CCT-GTAGCCCACGTCGTAGC-3′; *IL-1β* sense primer 5′-GAGATTGAGCTGTCTGCTCA-3′ and antisense primer 5′-AAGGAGAACCAAGCAACGAC-3′; and finally, *IL-6* sense primer 5′-GTACTCCAGAAGACCAGAGG-3′ and antisense primer 5′-TGCTGGTGACAA-CCACGGCC-3′ [53]. Glyceraldehyde 3-phosphate dehydrogenase (*Gapdh*) was amplified using the sense primer 5′-CTGGTGCTGAGTATGTCGTG-3′ and the antisense primer 5′-CAGTCTTCTGAGTGGCAGTG-3′ [54]. The PCR conditions were optimized for each set of primers. PCR products were analyzed on 1% agarose gels, stained with ethidium bromide, and visualized with a UV transilluminator. The optical density of the bands was analyzed using the NIH ImageJ software Fiji 1.53K, NIH, NY, USA (https://imagej.net/nih-image/, access date 2 July 2026). Bands for each cytokine mRNA were adjusted with the optical density values of the internal control, *Gapdh*, and expressed as a relative optical density (OD).

### 4.4. Evaluation of TNF-α, IL-1β, and IL-6 Levels in the Brains of Infected Mice

Total proteins were obtained from the OBs of infected and non-infected mice; brain tissues were incubated with 10 mL of extraction buffer [10 mM Tris (pH 7.4), 150 mM NaCl, 1% Triton X-100], which contained a cocktail of protease inhibitors [5 mM ethylenediaminetetraacetic acid (EDTA), 1 mM iodoacetamide (IAA), N-ethylmaleimide (NEM), 2 mM tosyl-L-lysyl-chloromethane hydrochloride (TLCK), 100 μg/mL phenylmethylsulfonyl fluoride (PMSF), and 0.225 U/mL aprotinin (all protease inhibitors from Sigma-Aldrich, St. Louis, MO, USA)] at 4 °C for 1 h. The samples were subsequently homogenized in a precooled sterile mortar with a pestle at 16,000× *g* for 20 min, after which the supernatants were collected and precipitated with acetone at 4 °C (J.T. Baker, Phillipsburg, NJ, USA) at a 9:1 ratio. After 1 h, the total proteins were centrifuged at 16,000× *g* for 30 min at 4 °C. The pellet was recovered and washed twice with extraction buffer without Triton X-100 (Sigma-Aldrich, St. Louis, MO, USA). The protein concentration was determined using a bicinchoninic acid (BCA) protein assay (Thermo Fisher Scientific, Waltham, MA, USA). For the Western blot assays, the protein concentration was adjusted to 10 μg for all the evaluated conditions. The proteins were resolved by SDS–PAGE using 4–20% gradient polyacrylamide gels at a constant voltage (90 V) for 3 h. Then, they were electrotransferred to Immunobilon-P (0.45 mm) polyvinylidene fluoride (PVDF) membranes (Merck Millipore, Darmstadt, HE, Germany) at 400 mA for 1 h at 4 °C. Subsequently, the PVDF membranes were blocked with 5% nonfat-dried milk (Sigma-Aldrich, St. Louis, MO, USA) in TBS (Tris-buffered saline, 50 mM Tris, 150 mM NaCl) supplemented with 0.05% Tween 20 for 1 h at room temperature. Later, they were incubated with primary rabbit polyclonal antibodies against IL-1β (Santa Cruz Biotechnology, Santa Cruz, CA, USA, sc-7884), IL-6 (Santa Cruz Biotechnology, Santa Cruz, CA, USA, sc-7920), and TNF-α (Santa Cruz Biotechnology, Santa Cruz, CA, USA, sc-8301). β-actin (Santa Cruz Biotechnology, Santa Cruz, CA, USA, sc-130656) was used as a loading control; it was dissolved in 5% nonfat dry milk in TBS-T and kept overnight at 4 °C. The membranes were incubated with anti-rabbit IgG labeled with HRP (Jackson ImmunoResearch, West Grove, PA, USA, 111–035–003; 1:5000), and the blots were revealed with a luminol kit reagent (Santa Cruz Biotechnology, Santa Cruz, CA, USA) using Kodak photographic film. The data are expressed as arbitrary units and were analyzed by densitometry. Densitometry was performed using the ImageJ program version 1.53K (https://imagej.net/nih-image/, access date 2 July 2026), and the results are expressed as the relative optical density (OD). All the data were analyzed by densitometry.

### 4.5. Detection of Inflammatory Cytokines by Immunohistochemistry

Immunohistochemistry assays were performed to analyze the production of proinflammatory cytokines at the site of infection. Briefly, the brains of infected animals were fixed using vascular perfusion with a 4% solution of paraformaldehyde diluted in phosphate-buffered saline (PBS; pH 7.2). Representative fragments of tissue were dissected and embedded in paraffin. Six micro-thick sections were stained with hematoxylin and eosin for light microscopic analysis. For the immunohistochemistry assays, we selected animals from 4, 5, and 7 days post-infection; to remove endogenous peroxidase, we used 8% H_2_O_2_ in methanol at room temperature for 1 h. Nonspecific reactions were blocked by treatment with 10% FBS for 1 h at room temperature. Sections were incubated overnight at 4 °C with rabbit polyclonal antibodies to detect TNF-α, IL-1β, and IL-6 (1:25 in 3% FBS; Santa Cruz Biotechnology, Santa Cruz, CA, USA). Rabbit preimmune serum was used as a control. The slides were subsequently washed 3 times for 5 min with PBS-Tween (0.05%), and the secondary antibody, goat anti-rabbit IgG labeled with peroxidase, was added (diluted 1:250; Sigma-Aldrich, St. Louis, MO, USA) and incubated for 2 h at room temperature. The slides were subsequently washed with PBS-Tween (0.05%), and the reaction was revealed with a diaminobenzidine kit (Pierce Biotechnology, Rockford, IL, USA). The samples were counterstained with hematoxylin and examined under a light microscope (Nikon Eclipse 80i, Tokyo, Japan).

### 4.6. Nitric Oxide Determination in Mouse Brains Infected with N. fowleri Trophozoites

To evaluate the NO production induced by *N. fowleri* during PAM progression, the levels of nitrite (NO_2_) and nitrate (NO_3_), which are the final products of NO, were determined. Briefly, tissue homogenates from 100 mg of tissue at different times post-infection were obtained in 500 μL of cool sterile PBS (pH 7.4) using Potter–Elvehjem-type tissue grinders with a PTFE pestle (Thomas Scientific, Swedesboro, NJ, USA). The total extracts were centrifuged at 10,000× *g* for 20 min, and the supernatants were deproteinized with Microcon centrifugal filters (10,000 MW; Millipore) in a centrifuge at 12,000 rpm for 30 min at 4 °C. The soluble fraction was recovered, and the NO concentrations were determined using a commercial nitrite/nitrate enzyme colorimetric assay kit (Cayman Chemical Co., Ann Arbor, MI, USA). This kit allows us to determine the final products of NO (NO_−2_ and NO_−3_), which together constitute the best index of total NO. After determining the nitrate and nitrite standard curve, the samples were processed following the manufacturer’s instructions. The data were obtained by spectrophotometry at 540 nm. The inter- and intra-assay percentages are estimated to be 3.4 and 2.7, respectively. Noninfected tissue from the brain was used to determine the concentration (μM) of NO_−2_ + NO_−3_, which was considered basal production. Statistical analysis (one-way ANOVA) was performed using SigmaPlot 12 software (http://www.sigmaplot.com, access date 2 July 2026). Graphs were constructed using GraphPad Prism 5 software. The data are expressed as the mean ± SEM of three independent experiments.

### 4.7. Detection of Reactive Oxygen Species During PAM Progression

To determine ROS production from 1 to 7 days of PAM evolution, the brains of 100 mg of infected mouse tissue were obtained and treated with fluorescent reactive 2′,7′-Dichlorodihydrofluorescein diacetate (DCFH-DA, Sigma-Aldrich, St. Louis, MO, USA) in RPMI medium at 37 °C for 30 min with slight agitation. The tissue samples were homogenized using a Potter homogenizer with 2 mL of cool PBS (pH 7.4). The fluorescence of DCFH-DA was measured using a Perkin-Elmer LS50B fluorescence spectrophotometer (excitation wavelength, 485 nm; emission wavelength, 525 nm) [55]. Brain samples from healthy mice were incubated with 1 mM H_2_O_2_ for 30 min, which was used as a positive control for oxidative stress. The data were compared with the relative fluorescence intensity (RFI) of healthy controls. The data are expressed as the mean ± SEM of three independent experiments.

### 4.8. Statistical Analysis

ANOVA was used for global statistics, and Dunnett’s multiple-comparison test and the Bonferroni test were used to compare treated groups vs. the control group and all groups, respectively. *p* < 0.05 was considered statistically significant.

## Figures and Tables

**Figure 1:**
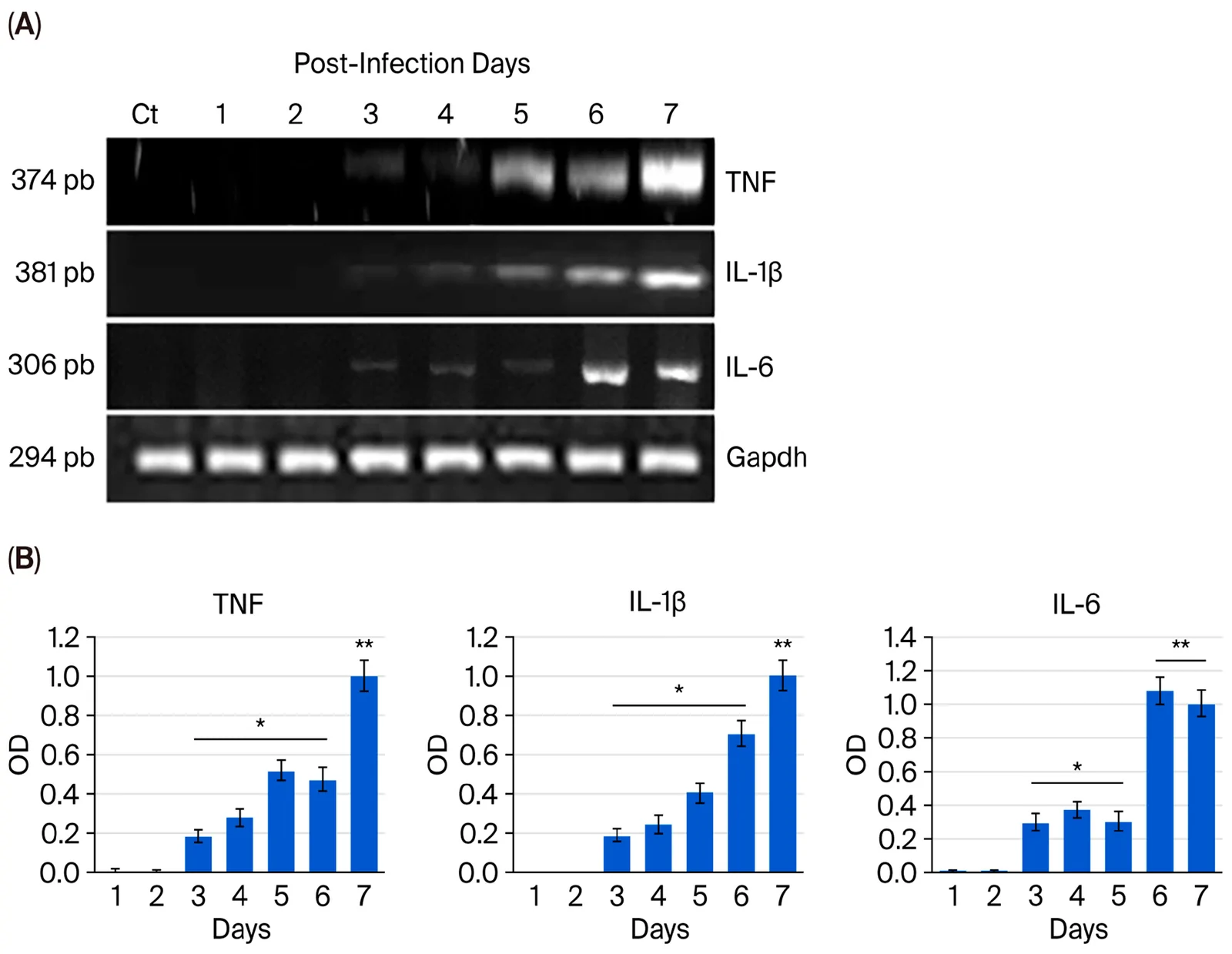
*N. fowleri* induces the mRNA expression of *TNF*, *IL-1β*, and *IL-6* in the mouse brain: (**A**) RT–PCR assays for *TNF*, *IL-1β*, and *IL-6* from infected animals at 1, 2, 3, 4, 5, 6, and 7 days post-infection. Healthy animals were used as the experimental control (Ctrl). (**B**) Densitometry analysis of each inflammatory cytokine was performed with ImageJ software version 1.53K (Wayne Rasband and contributors, NIH, Java 13.06). Relative optical density (OD) values were normalized to those of the housekeeping gene *Gapdh* and are expressed as the mean ± SD of three independent assays; * *p* < 0.05; ** *p* < 0.01.

**Figure 2:**
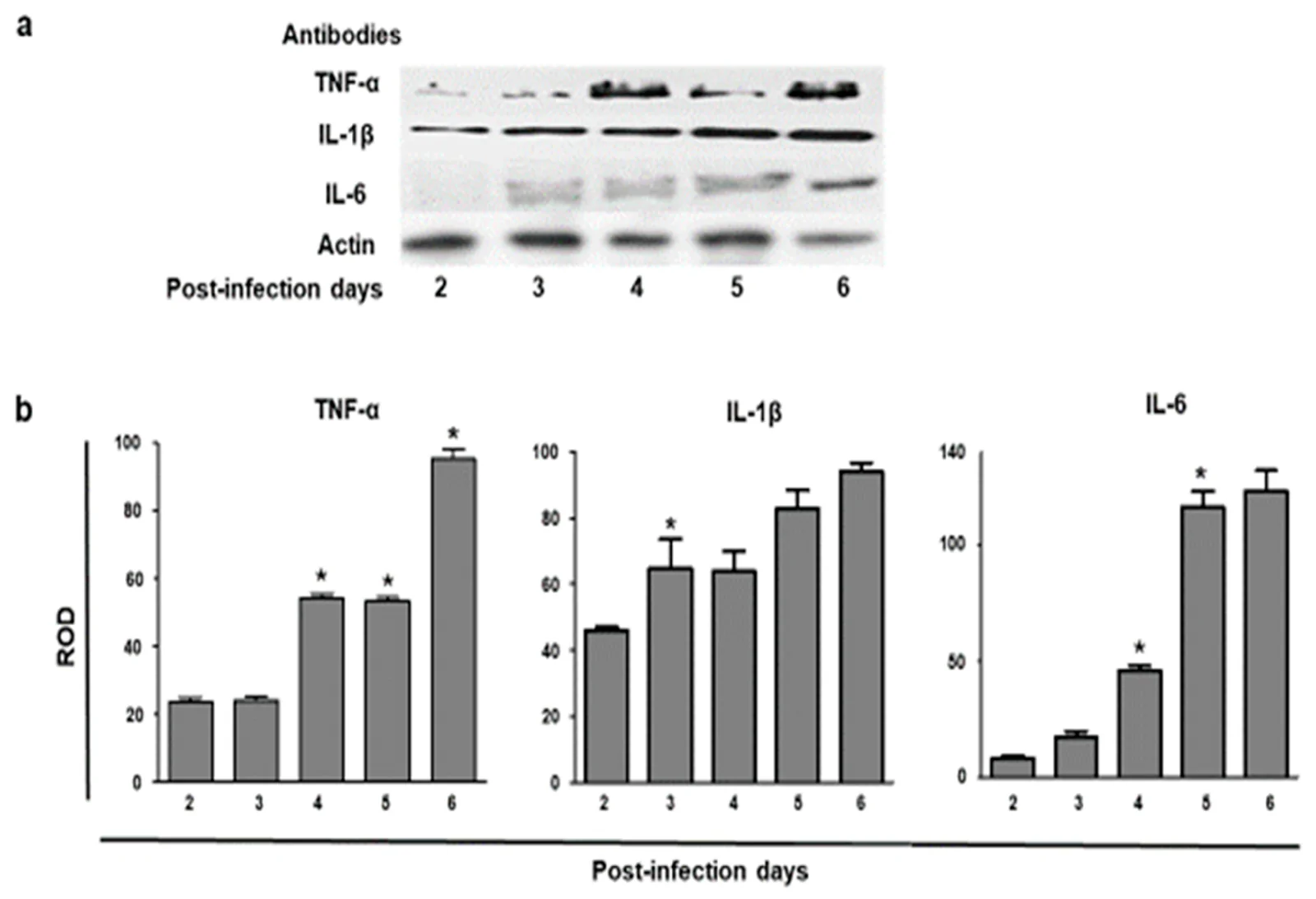
Production of TNF-α, IL-1β, and IL-6 during experimental PAM: (**a**) Western blot analysis of TNF-α, IL-1β, and IL-6 production at 2, 3, 4, 5, and 6 days post-infection revealed that the overproduction of all cytokines evaluated occurred in a time-dependent manner. Actin protein was used as a loading control. (**b**) Densitometry analysis of the cytokines mentioned above was performed using ImageJ software, and the results are expressed as OD values. The data were normalized to those of the actin protein and are presented as the mean ± SD of three independent assays; * *p* < 0.05.

**Figure 3:**
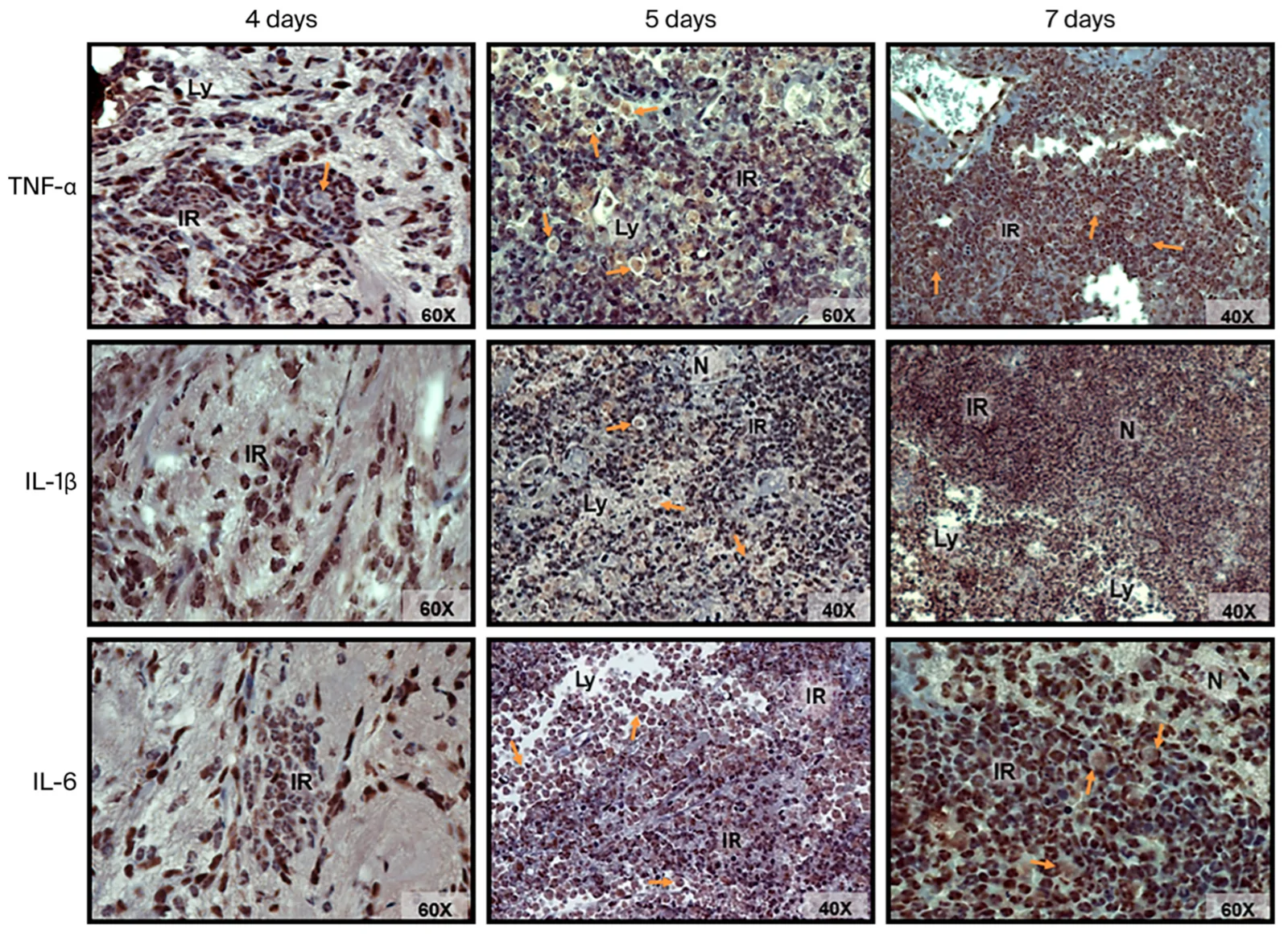
In situ production of proinflammatory cytokines induced by *N. fowleri*. Representative immunohistochemistry samples show TNF-α-, IL-1β-, and IL-6-positive cells, which were mainly observed during the inflammatory reaction (IR) at 4 days post-infection. The inflammatory response increased markedly at days 5 and 6 in the presence of *N. fowleri* trophozoites (orange arrows), and lytic (Ly) and necrotic (N) areas were observed in the mouse olfactory bulbs.

**Figure 4:**
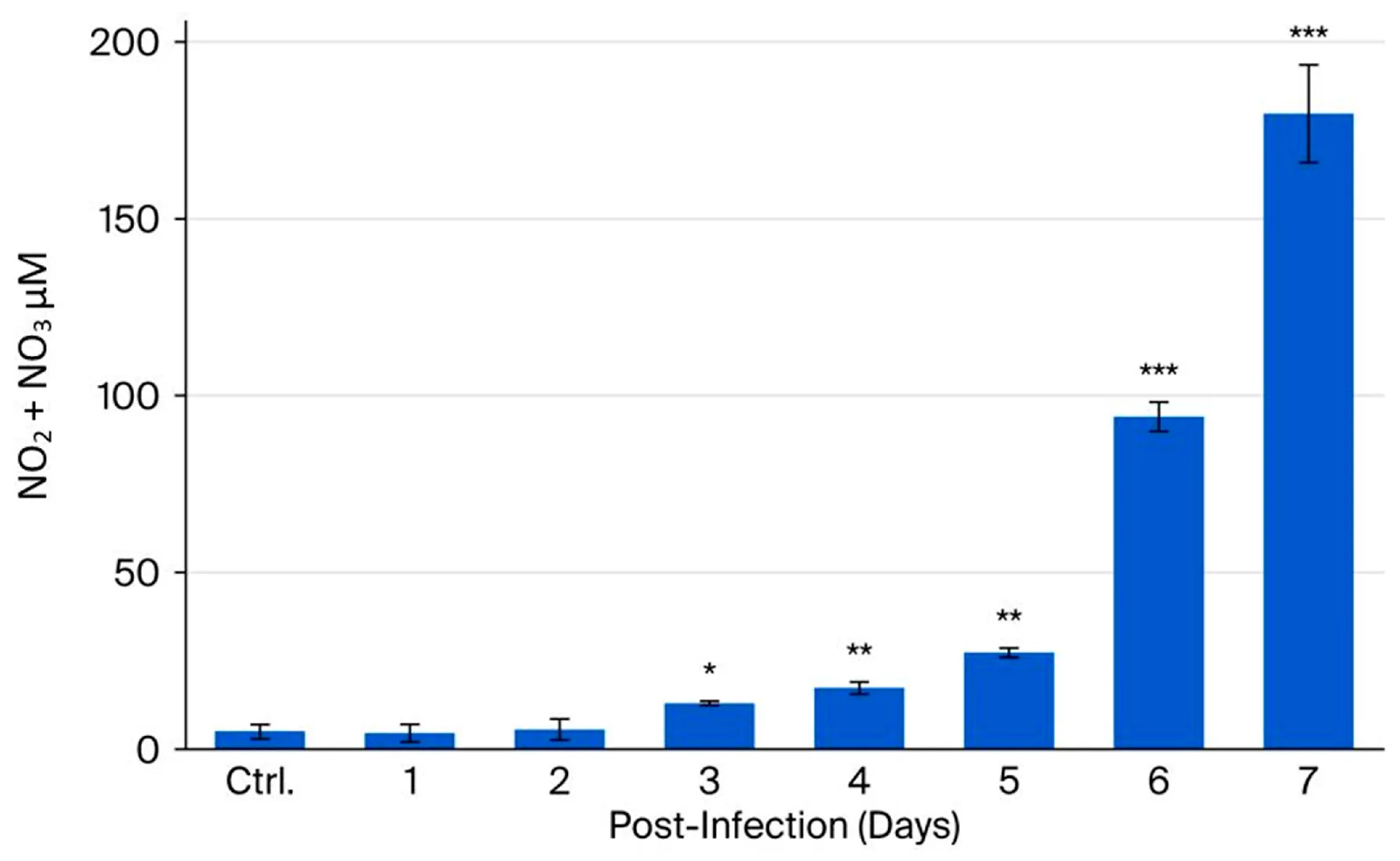
Nitric oxide production during PAM progression. The production of NO_2_ + NO_3_ in the mouse brains was measured from the first day to the seventh day post-infection. The results of the statistical analysis of the spectrophotometric measurements revealed that the values increased significantly after the third day, with a peak on the seventh day post-infection, compared with those in the brains of healthy mice (Ctrl). The concentration of NO_2_ + NO_3_ (μM) was compared and normalized to the basal production of healthy animals and is presented as the mean ± SD of three independent assays; * *p* < 0.05, ** *p* < 0.01, *** *p* < 0.001.

**Figure 5:**
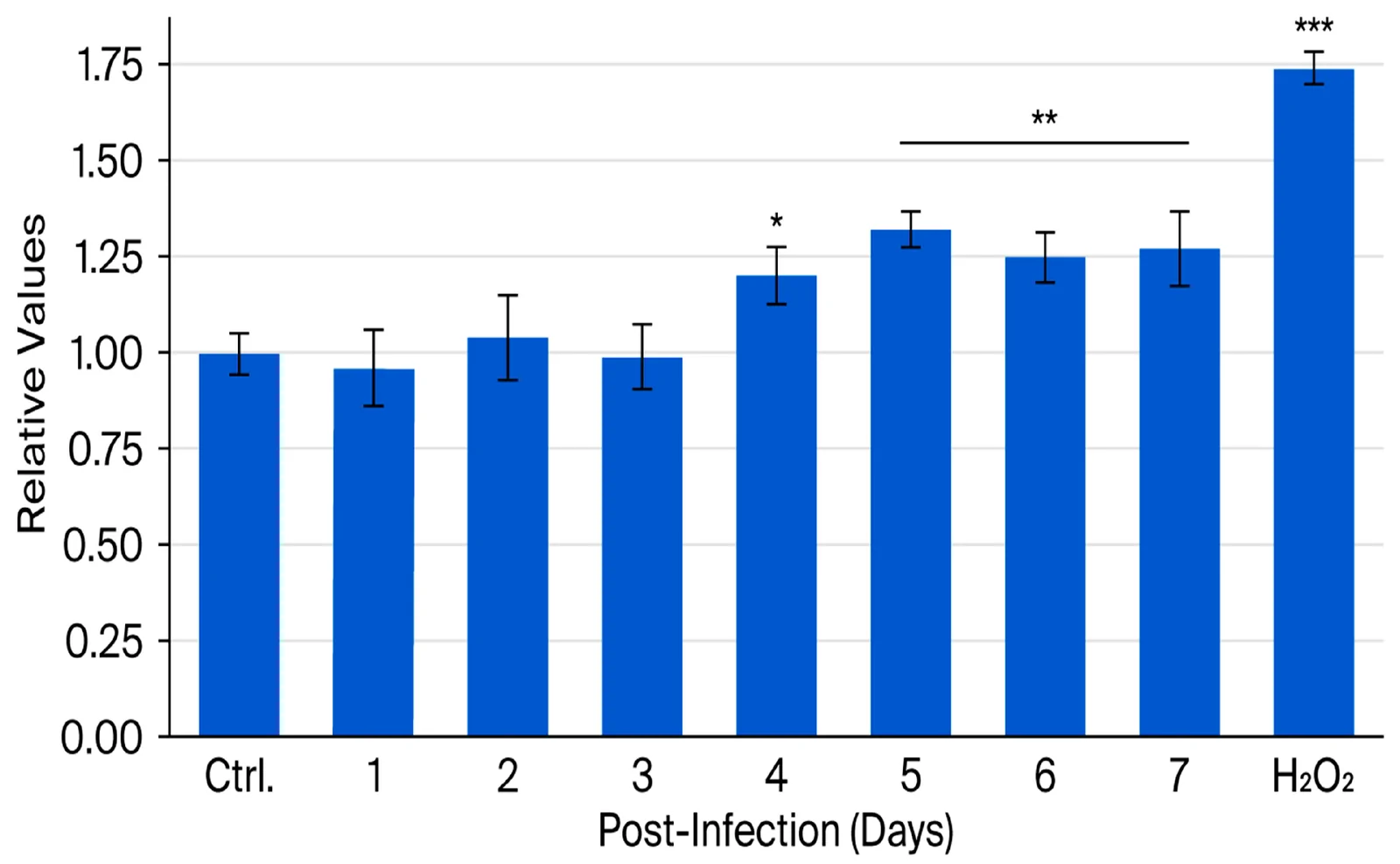
Reactive oxygen species production in mice infected with *N. fowleri*. ROS production in the brain increased significantly from days four to seven post-infection. The level of ROS production induced by *N. fowleri* was compared to the level of basal ROS production in noninfected animals (Ctrl). Brains from healthy mice were incubated with H_2_O_2_ as a positive control for ROS production. The spectrofluorometric values were adjusted and normalized to the basal production of healthy animals and are presented as the mean ± SD of three independent assays; * *p* < 0.05, ** *p* < 0.01, *** *p* < 0.001.

## Data Availability

The original contributions presented in this study are included in the article. Further inquiries can be directed to the corresponding author.

## Abbreviations

The following abbreviations are used in this manuscript:

PAM	Primary amoebic meningoencephalitis
NO	Nitric oxide
ROS	Reactive oxygen species
CNS	Central nervous system
OB	Olfactory bulb
NOS	Nitric oxide synthase
NETs	Neutrophil extracellular traps
